# Supplementary material for: Content-rich biological network constructed by mining PubMed abstracts
Source: BMC Bioinformatics. 2004 Oct 8;5:147. doi: 10.1186/1471-2105-5-147 (PMC528731; doi:10.1186/1471-2105-5-147)
Supplement: Additional File 2 — The original results of the above study (non-essential files are deleted to keep the file size under the limit set by BMC bioinformatics). [file 1471-2105-5-147-S2.bz2 › chilibotAdditionalFile2/dip50/601ID10052460E10980/html/HOXB1_PBX1.html]

 


 **HOXB1** and **PBX1** 
  
Found 178 abstracts in PubMed, retrieved 50.  
 

 What does Google say? 
 PDF only 
| .edu only 

---

**Interactive relationship** (e.g. stimulation, inhibition, etc)

**Stimulatory relationship**- A bipartite Hox Pbx  [ **PBX1** ]  binding motif is located within this enhancer, and in vitro DNA binding experiments showed that the vertebrate labial related protein  **Hoxb1**  will cooperatively bind to this site in a Pbx  [ **PBX1** ]  Exd dependent manner.  Ref: 9242495 Genes Dev, 1997
- Notable in this context is the homeobox  [ **PBX1** ]  gene HOX B8 formerly known as HOX 2  [ **HOXB1** ] .4, which was shown to cooperate with hematokines to induce leukemia, and to enhance self renewal of immature myeloid progenitors when expressed alone.  Ref: 9292516 Blood, 1997
- However, on the  **hoxb1**  autoregulatory enhancer, Hoxa1 and Hoxa1 NA KR may display distinct activity despite the presence of Pbx  [ **PBX1** ] , in a cell type dependent manner.  Ref: 12060683 Nucleic Acids Res, 2002
- The r4 enhancers from  **Hoxb1**  and Hoxb2 have the same activity, but differ with respect to the number and organisation of bipartite Pbx  [ **PBX1** ]  Hox PH sites required, suggesting the intervention of other components sequences.  Ref: 10654609 Development, 2000
**Neutral relationship**- Interaction with Prep1 enhances the ability of the  **HOXB1**   **Pbx1**  complex to activate transcription in a cooperative fashion from the same target.  Ref: 9482740 EMBO J, 1998
- In the presence of all three nuclear factors, cooperative interactions between recombinant  **PBX1**  and PREP1 or  **PBX1**  and  **HOXB1**  result in binding of the heterodimers to FPB in vitro.  Ref: 10748126 J Biol Chem, 2000
- DNA binding assays and cell transfection experiments revealed that the bipartite core sequence of FPB 5 ATCAATCA 3 binds the  **PBX1**  2, PREP1, and  **HOXB1**  proteins, and this in turn leads to promoter transactivation.  Ref: 10748126 J Biol Chem, 2000
- The DNA binding and protein protein interaction functions of  **HOXB1**  and  **Pbx1**  are all necessary for the assembly of a transcriptionally active complex on the b1 ARE.  Ref: 9218805 EMBO J, 1997
- This observation implies that this region is at least partially preformed and thus ready to interact with  **Pbx1**  and stabilize binding of  **Pbx1**  and  **HoxB1**  to DNA.  Ref: 11369863 Protein Sci, 2001
- Conformational changes induced in Hoxb  [ **HOXB1** ]  8 Pbx 1  [ **PBX1** ]  heterodimers in solution and upon interaction with specific DNA.  Ref: 9271414 Mol Cell Biol, 1997
- On the basis of these experiments we conclude that the r4 restricted domain of Hoxb2 in the hindbrain is the result of a direct cross regulatory interaction by  **Hoxb1**  involving vertebrate Pbx  [ **PBX1** ]  proteins as cofactors.  Ref: 9242495 Genes Dev, 1997
- Furthermore, upon interaction with specific DNA, additional conformational changes are induced in the Pbx 1  [ **PBX1** ]  Hoxb  [ **HOXB1** ]  8 heterodimer.  Ref: 9271414 Mol Cell Biol, 1997
- The  **Hoxb1**  autoregulatory element comprises three HOX PBX  [ **PBX1** ]  binding sites.  Ref: 11046157 Mol Cell Biol, 2000
- The  **HoxB1**  hexapeptide is a prefolded domain implications for the  **Pbx1**  Hox interaction.  Ref: 11369863 Protein Sci, 2001
- As a consequence,  **Hoxb1**  employs Pbx  [ **PBX1** ]  and Meis related proteins, as a pair of essential cofactors in a higher order molecular complex, to mediate its transcriptional effects on an endogenous Hox response element.  Ref: 10373562 Mol Cell Biol, 1999
- Hoxb  [ **HOXB1** ]  5 is one of the few homeobox  [ **PBX1** ]  genes strongly expressed in the developing mouse lung.  Ref: 9450632 Histochem Cell Biol, 1997
- This element contains multiple Hox Pbx  [ **PBX1** ]  consensus binding sites that bind to both HOXA1  **Pbx1**  and  **HOXB1**   **Pbx1**  proteins in vitro.  Ref: 9733765 J Biol Chem, 1998
- The murine Hoxb 1  [ **HOXB1** ]  gene contains a homeobox  [ **PBX1** ]  sequence and is expressed in a spatiotemporal specific pattern in neuroectoderm, mesoderm and gut endoderm during development.  Ref: 9671595 Development, 1998
- Like PREP1, PREP2 PBX  [ **PBX1** ]  heterodimers are capable of forming ternary complexes with  **HOXB1** .  Ref: 11972344 Nucleic Acids Res, 2002
- The crystal structure of a human  **HoxB1**   **Pbx1**  DNA ternary complex revealed that interactions between the two proteins are mediated by the  **HoxB1**  hexapeptide, which inserts into a hydrophobic pocket in  **Pbx1** .  Ref: 10448033 J Mol Biol, 1999
- Three members of the Antennapedia family, Hoxc 6, Hoxb  [ **HOXB1** ]  7, and Hoxb  [ **HOXB1** ]  8, select the same binding site in conjunction with  **Pbx1** .  Ref: 9234726 Mol Cell Biol, 1997
- NMR studies of the  **pbx1**  TALE homeodomain protein free in solution and bound to DNA proposal for a mechanism of  **HoxB1**   **Pbx1**  DNA complex assembly.  Ref: 10448033 J Mol Biol, 1999
- Heterodimer contacts are mediated by the hexapeptide of  **HoxB1** , which binds in a pocket in the  **Pbx1**  protein formed in part by a three amino acid insertion in the  **Pbx1**  homeodomain.  Ref: 10052460 Cell, 1999
- Herein we describe NMR structural calculations of the hexapeptide of  **HoxB1**  Nalpha acetyl Thr Phe Asp Trp Met Lys amide that has been shown to mediate binding between  **HoxB1**  and  **Pbx1**  and a hexapeptide consensus sequence Nalpha acetyl Leu Phe Pro Trp Met Arg amide.  Ref: 11369863 Protein Sci, 2001
- The structures of the  **HoxB1**  peptide and the trans form of the consensus peptide reveal a turn very similar to that found as part of the  **HoxB1**   **Pbx1**  DNA complex in the X ray crystal structure.  Ref: 11369863 Protein Sci, 2001
- Binding site selection for Hoxb  [ **HOXB1** ]  9 with Pbx1a yielded ATGATTTACGAC, containing a novel TTAC Hox binding site adjacent to a Pbx  [ **PBX1** ]  site.  Ref: 9079637 J Biol Chem, 1997
- Human PREP1, a novel homeodomain protein of the TALE super family, forms a stable DNA binding complex with PBX  [ **PBX1** ]  proteins in solution, a ternary complex with PBX  [ **PBX1** ]  and  **HOXB1**  on DNA, and is able to act as a co activator in the transcription of PBX  [ **PBX1** ]   **HOXB1**  activated promoters Berthelsen, J.  Ref: 10381567 Mech Dev, 1999
- The  **Pbx1**  DNA binding domain is larger than the canonical homeodomain, containing an additional alpha helix that appears to contribute to binding of the  **HoxB1**  hexapeptide and to stable binding of  **Pbx1**  to DNA.  Ref: 10052460 Cell, 1999
- PREP1 is able to super activate the PBX  [ **PBX1** ]  HOXB 1  [ **HOXB1** ]  autoregulated Hoxb 1  [ **HOXB1** ]  promoter, and we show that all three proteins, PREP1, PBX  [ **PBX1** ]  and HOXB 1  [ **HOXB1** ] , are present together in the mouse rhombomere 4 domain in vivo, compatible with a role of PREP1 as a regulator of PBX  [ **PBX1** ]  and HOXB 1  [ **HOXB1** ]  proteins activity during development.  Ref: 10381567 Mech Dev, 1999

**Non-interactive relationship** (e.g. studied together, co-existance, homology, etc.)

- In this study, we demonstrated that Pbx  [ **PBX1** ]  and Meis bind DNA as heterotrimeric complexes with  **Hoxb1**  on a genetically defined Hoxb2 enhancer, r4, that mediates the cross regulatory transcriptional effects of  **Hoxb1**  in vivo.  Ref: 10373562 Mol Cell Biol, 1999
- In contrast, no change in conformation is observed upon mixing the  **HoxB1**  protein with  **Pbx1**  in the absence of DNA.  Ref: 10448033 J Mol Biol, 1999
- Analyses of the expression and targeted disruption of the homeobox  [ **PBX1** ]  gene  **Hoxb1**  demonstrate that it is essential for patterning progenitor cells along the entire DV axis of rhombomere 4 r4.  Ref: 11076756 Development, 2000
- Analysis of two distinct retinoic acid response elements in the homeobox  [ **PBX1** ]  gene  **Hoxb1**  in transgenic mice.  Ref: 11891985 Dev Dyn, 2002
- Structure of a  **HoxB1**   **Pbx1**  heterodimer bound to DNA role of the hexapeptide and a fourth homeodomain helix in complex formation.  Ref: 10052460 Cell, 1999
- Co expression of either HOXA1 or  **HOXB1**  with  **Pbx1**  transactivated EphA2 enhancer dependent reporter gene expression.  Ref: 9733765 J Biol Chem, 1998
- This study suggests a model for the assembly of a stable  **HoxB1**   **Pbx1**  DNA ternary complex.  Ref: 10448033 J Mol Biol, 1999
- The methods proposed in this paper are then applied to population data at the dopamine D2 receptor locus DRD2 and at the homeobox  [ **PBX1** ]  B HOXB  [ **HOXB1** ]  gene cluster.  Ref: 10738528 Ann Hum Genet, 1999
- Mice were generated with a targeted disruption of the homeobox  [ **PBX1** ]  containing gene hoxb  [ **HOXB1** ]  9.  Ref: 9013929 Dev Biol, 1997
- Therefore, we hypothesize that expression of two additional mesenchymally derived nuclear transcription factors, retinoid X receptor alpha RXR alpha and homeobox  [ **PBX1** ]  b 5 Hoxb  [ **HOXB1** ]  5, would also be altered in hypoplastic lungs.  Ref: 12505043 J Surg Res, 2002
- Several homeobox  [ **PBX1** ]  genes of the HOXA and HOXB  [ **HOXB1** ]  clusters are expressed in primitive blood cells, suggesting a role for HOX genes in normal hematopoiesis.  Ref: 9058712 Blood, 1997
- The strong repressive effect of RIP140 was restricted to glucocorticoid mediated responses in as much as it slightly increased signaling through the RelA and the Pit 1 Pbx  [ **PBX1** ]  proteins and only slightly repressed signaling through the  **Pbx1**   **HOXB1**  and AP 1 proteins, excluding general squelching as a mechanism.  Ref: 10364267 J Biol Chem, 1999
- We demonstrate that RIP140 antagonized all GR mediated responses tested, which included activation through classical GRE, the synergistic effects of glucocorticoids on AP 1 and  **Pbx1**   **HOXB1**  responsive elements, as well as gene repression through a negative GRE and cross talk with NF kappaB RelA.  Ref: 10364267 J Biol Chem, 1999
- A genetic polymorphism in the human  **HOXB1**  homeobox  [ **PBX1** ]  gene implying a 9bp tandem repeat in the amino terminal coding region.  Ref: 10671062 Hum Mutat, 1998
- Indeed, we observe the formation of a ternary Prep1  **Pbx1**   **HOXB1**  complex on a  **HOXB1**  responsive target in vitro.  Ref: 9482740 EMBO J, 1998
- We previously showed that  **HOXB1**  and  **PBX1**  activate transcription from the b1 ARE by binding to sequences required for the expression of a reporter gene in rhombomere 4 in vivo.  Ref: 11278854 J Biol Chem, 2001
- The authors previously have shown that the homeobox  [ **PBX1** ]  gene Hoxb  [ **HOXB1** ]  5 is necessary for normal airway branching during lung development.  Ref: 11101743 J Pediatr Surg, 2000
- Disruption of the homeobox  [ **PBX1** ]  gene Hoxb  [ **HOXB1** ]  6 in mice results in increased numbers of early erythrocyte progenitors.  Ref: 10996827 Am J Hematol, 2000
- We have examined how the expression of Xenopus homeobox  [ **PBX1** ]  gene, Hoxb 1  [ **HOXB1** ] , is regulated by instruction from the mesoderm and or endoderm and ask whether this instruction is by the vertical or planar routes.  Ref: 9133441 Dev Biol, 1997
- We report that the human  **Pbx1**  and  **HOXB1**  proteins can cooperatively activate transcription through a genetically characterized Hox target, i.e. an autoregulatory element directing spatially restricted expression of the murine Hoxb 1  [ **HOXB1** ]  gene b1 ARE in the developing hindbrain.  Ref: 9218805 EMBO J, 1997
- We show that, in P19 embryonal carcinoma cells, GCs potentiate RA induced expression of the murine Hoxb 1  [ **HOXB1** ]  gene through an autoregulatory element, b1 ARE, recognized by the  **Pbx1**  and  **HOXB1**  homoeodomain proteins.  Ref: 12487626 Biochem J, 2003
- In man there are 39 homeobox  [ **PBX1** ]  genes of the HOX family in four loci, HOXA, HOXB  [ **HOXB1** ] , HOXC and HOXD on chromosomes 2, and 2.  Ref: 10671062 Hum Mutat, 1998
- Despite the presence of  **HOXB1**  and  **PBX1** , this enhancer fails to activate reporter gene expression in retinoic acid treated P19 cell monolayers.  Ref: 11046157 Mol Cell Biol, 2000
- On the b1 ARE, only a restricted subset of HOX proteins HOXA1,  **HOXB1** , HOXA2 are able to bind cooperatively with  **Pbx1**  and activate transcription.  Ref: 9218805 EMBO J, 1997
- An enhancer element in the EphA2 Eck gene sufficient for rhombomere specific expression is activated by HOXA1 and  **HOXB1**  homeobox  [ **PBX1** ]  proteins.  Ref: 9733765 J Biol Chem, 1998
- Functional dissection of the complex allowed the localization of the main activation domain in the  **HOXB1**  N terminal region, and of an additional one in the C terminal region of  **Pbx1**  contained in the Pbx1a but not in the alternatively spliced Pbx1b isoform.  Ref: 9218805 EMBO J, 1997
- Similarly, overexpression of different combinations of  **PBX1** , PREP1, and  **HOXB1**  transactivates FPB driven transcription.  Ref: 10748126 J Biol Chem, 2000
- We report here the 2.35 A structure of a ternary complex containing a human  **HoxB1**   **Pbx1**  heterodimer bound to DNA.  Ref: 10052460 Cell, 1999
- Association of bronchopulmonary sequestration with expression of the homeobox  [ **PBX1** ]  protein Hoxb  [ **HOXB1** ]  5.  Ref: 11101743 J Pediatr Surg, 2000
- Additional transfection experiments showed that the  **Pbx1**   **HOXB1**  heterodimer was the target for the GC effect.  Ref: 12487626 Biochem J, 2003
- These results, together with observations of reduced EphA2 expression in hoxa1 and  **hoxb1**  double mutant mice, suggest that expression of EphA2 gene in rhombomere 4 is directly regulated by Hoxa1 and  **Hoxb1**  homeobox  [ **PBX1** ]  transcription factors.  Ref: 9733765 J Biol Chem, 1998
